# Changes in miRNA expression in the lungs of pigs supplemented with different levels and forms of vitamin D

**DOI:** 10.1007/s11033-023-08940-1

**Published:** 2023-12-12

**Authors:** Alicja Wierzbicka, Klaudia Pawlina-Tyszko, Małgorzata Świątkiewicz, Tomasz Szmatoła, Maria Oczkowicz

**Affiliations:** 1 Department of Animal Molecular Biology, National Research Institute of Animal Production, Ul. Krakowska 1, Balice, 32-083 Poland; 2 Department of Animal Nutrition and Feed Science, National Research Institute of Animal Production, Ul. Krakowska 1, Balice, 32-083 Poland; 3 Center for Experimental and Innovative Medicine, University of Agriculture in Kraków, Rędzina 1c, Kraków, 30 248 Poland

**Keywords:** Cholecalciferol, Calcidiol, Lungs, miRNA-seq, Swine

## Abstract

**Background:**

Vitamin D is an immunomodulator, and its effects have been linked to many diseases, including the pathogenesis of cancer. However, the effect of vitamin D supplementation on the regulation of gene expression of the lungs is not fully understood. This study aims to determine the effect of the increased dose of cholecalciferol and a combination of cholecalciferol + calcidiol, as well as the replacement of cholecalciferol with calcidiol, on the miRNA profile of healthy swine lungs.

**Methods and results:**

The swine were long-term (88 days) supplemented with a standard dose (2000IU/kg) of cholecalciferol and calcidiol, the increased dose (3000 IU/kg) of cholecalciferol, and the cholecalciferol + calcidiol combination: grower: 3000 IU/Kg of vitamin D (67% of cholecalciferol and 33% of calcidiol), finisher 2500 IU/Kg of vitamin D (60% of cholecalciferol and 40% of calcidiol). Swine lung tissue was used for Next Generation Sequencing (NGS) of miRNA. Long-term supplementation with the cholecalciferol + calcidiol combination caused significant changes in the miRNA profile. They embraced altered levels of the expression of miR-150, miR-193, miR-145, miR-574, miR-340, miR-381, miR-148 and miR-96 (*q-value* < 0.05). In contrast, raising the dose of cholecalciferol only changed the expression of miR-215, and the total replacement of cholecalciferol with calcidiol did not significantly affect the miRNAome profile.

**Conclusions:**

The functional analysis of differentially expressed miRNAs suggests that the use of the increased dose of the cholecalciferol + calcidiol combination may affect tumorigenesis processes through, inter alia, modulation of gene regulation of the TGF- β pathway and pathways related to metabolism and synthesis of glycan.

**Supplementary Information:**

The online version contains supplementary material available at 10.1007/s11033-023-08940-1.

## Introduction

Vitamin D_3_ (cholecalciferol), also known as the sun vitamin, is currently one of the most recommended dietary supplements for improving general health in humans and animals.

It is assessed that the vitamin D receptor (VDR) regulates up to 3% of genes in both the human and the mouse genomes [1]. Vitamin D affects *VDR* expression and can influence epigenetic processes by regulating miRNA expression [2]. Interestingly, although the expression of the enzyme CYP27B1 responsible for converting vitamin D into its active form is most pronounced in the kidney, its production is observed in many other tissues including the lungs, suggesting the possibility of local activation of this vitamin [3].

In recent years, interest in vitamin D has increased because there have been many reports of its extra-skeletal effects, e.g. on the immune system. Strengthening the immunity of animals is currently one of the priorities, therefore assessing the effects of vitamin D supplementation on changes in the lungs, which is the repertoire of tissue-resident immune immune cells, seems to be crucial. The domestic pig is an animal of great economic importance around the world. At the same time, it is considered a very good model animal due to its similar physiology and size of main organs to humans. Therefore, our experiment, on the one hand, assessed the validity of using an increased dose and various forms of vitamin D to improve the functioning of the respiratory system of animals and, on the other hand, allowed us to trace the impact of these modifications on the miRNAome of lungs in the context of lung diseases in humans.

Vitamin D exerts antibacterial and anti-inflammatory effects, as well as plays an antioxidant function [4]. Nevertheless, knowledge about the effect of vitamin D supplementation on the health of the respiratory system in farm animals is scarce, despite that they are often exposed to dust, what makes them prone to respiratory diseases. Contrary, in humans, vitamin D deficiencies are linked to several lung diseases such as acute lung injury, asthma, pneumonia, cystic fibrosis, pulmonary fibrosis, tuberculosis, and COPD (chronic obstructive pulmonary disease) [3]. Due to its significance in inflammatory mechanisms, vitamin D has emerged as a potential therapeutic agent in patients with chronic and acute respiratory diseases [4]. Interestingly, both association and clinical trial studies demonstrated that vitamin D concentration in blood serum is closely related to the course of COVID-19 disease [5]. Moreover, the results of one randomized controlled trial in the field of oncology indicated that vitamin D_3_ supplementation can improve the survival of patients with early-stage lung cancer [6]. It has also been proven that the chemopreventive effects of vitamin D are associated with the regulation of tumour suppressor miRNAs (miR-100 and miR-125b) [2].

Recently, there has been a lot of discussion about the need to update the recommendations concerning the dosage and supplementation of calcidiol (calcifediol) in humans [7, 8]. According to some researchers, current recommendations for vitamin D intake are adequate for skeletal disorders, while for extra-skeletal disorders treatment much higher vitamin D doses are needed. It is also known that high-dose vitamin D supplementation is effective and well tolerated by the body, which is supported by randomized control trials of high-risk COVID-19 patients [9, 10]. In pigs, the maximum dose of vitamin D in the feedstuff is 2000IU/Kg, according to the European Union regulations ref. However, in the light of current knowledge, the question arises whether these recommendations should not be changed. Moreover, attempts to replace traditional cholecalciferol supplementation with calcidiol seem to be important for this issue. This is evidenced by our previous study, but also the findings of other researchers, indicate that calcidiol supplementation is more effective and faster in increasing 25(OH)D serum levels than cholecalciferol supplementation in pigs and in humans [11–13].

Although the effects of vitamin D have been researched for many years, the effect of cholecalciferol and calcidiol supplementation on the whole miRNAome has not been studied yet. It is well known that miRNAs, which belong to the class of short non-coding-RNAs, act as post-transcriptional regulators. They bind complementary mRNAs and prevent further protein synthesis. It is recognised that miRNA sequences are involved in the regulation of more than 60% of protein-coding genes [14]. Thus, the analysis of changes in the global miRNA profile can provide data which either confirms previous findings or points to new directions in the study of the effects of vitamin D.

## Materials and methods

### Animals and diets

All procedures on live animals included in this study received consent from the local Ethics Committee for Experiments with Animals in Cracow, Poland (Resolution No. 427/2020 of 22.07.2020). The animals were kept in individual straw-bedded pens at the Research Station of the National Research Institute of Animal Production in Grodziec Śląski. Swine were randomly divided into four dietary groups but all the animals were kept in the same environmental conditions. Group 1, *n* = 12 individuals, group 2, *n* = 12, group 3, *n* = 12, and group 4, *n* = 12. Each group included an equal number of males and females, except group 2 (3 females and 4 males). The male swine had previously been castrated.

**Fig. 1:**
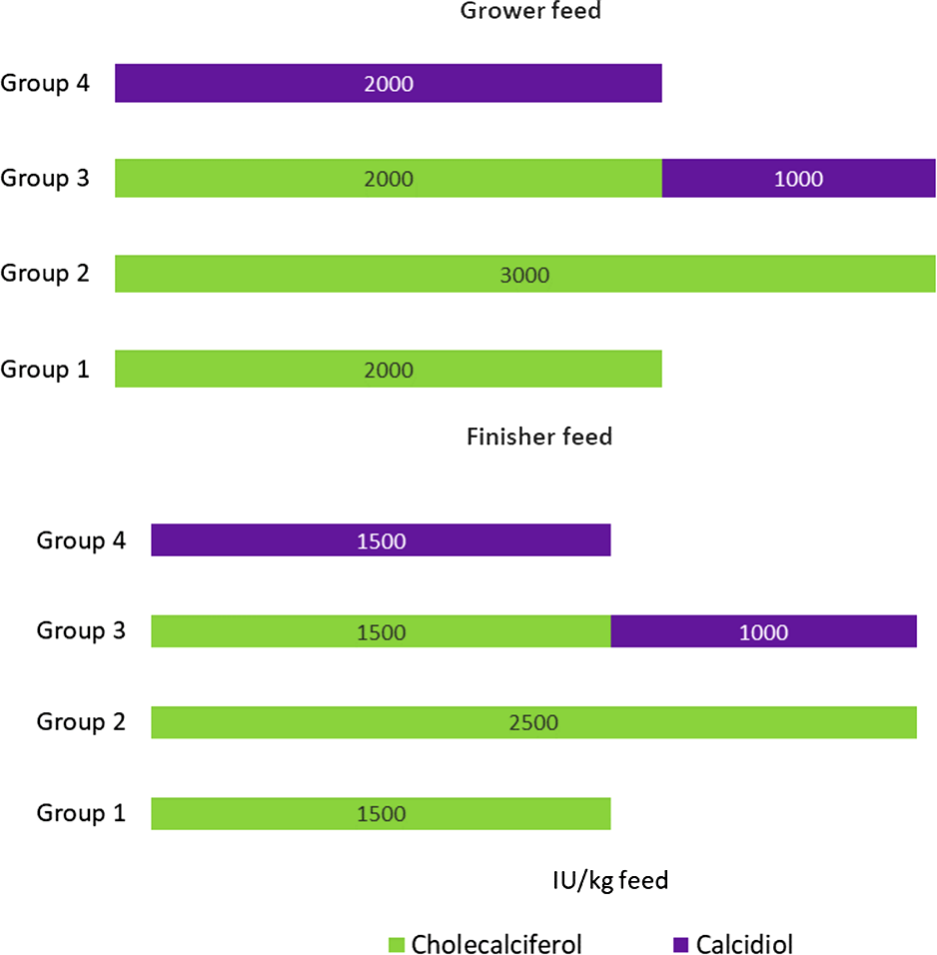
The content of cholecalciferol and calcidiol in the grower and finisher feeds

The diets of animals differed from each other only in the dose and form of vitamin D (Fig. 1.). In this experiment, animals receiving a standard dose (grower: 2000 IU/Kg, finisher: 1500 IU/Kg) of cholecalciferol in feed constituted the control group. The diets used were isonitrogenous and isoenergetic, and they covered all nutritional requirements of the swine (grower: metabolizable energy − 13.3 MJ, total protein − 172 g/kg; finisher: metabolizable energy − 13.3 MJ, total protein − 156 g/kg) [15]. The diet was determined by the needs of the animals and accounted for their current age and weight (grower − 30-60 kg, finisher − 60-110 kg).

After 88 days, when the animals reached a weight of ~ 110 kg, the feeding experiment ended, and 48 lung samples were collected from the animals. All the samples came from the middle part of the upper lobe of the left lung. Each swine was slaughtered using stunning high-voltage electric tongs (voltage 240–400 V). Immediately after, the lung samples were placed in a ULT freezer (− 85 °C) and stored until the miRNA analysis.

During slaughter, animals’ blood was collected into tubes with anticoagulant (acid dextrose citrate/glucose from the animals. The blood was used to assess serum vitamin D concentrations. The blood samples were then transported at + 6 °C to the laboratory, where blood plasma was obtained by centrifugation at 3000 RPM. The plasma samples were stored at -20.

### Measurement of 25(OH)D concentration

The measurement was conducted for 8 animals from each group. Assessment of total plasma vitamin D concentration was performed by ANCHEM Laboratorium, 20 Fredry Street, Katowice, Poland. The blood plasma samples were transported to the laboratory in a frozen state. The assessment was performed by RIA method. The DIAsource 25OH Vitamin D total-RIA-CT Kit (Rue de Bosquet 2, 1348 Louvain-La-Neuve, Belgium) and Multigamma 1260 multidetector instrument (LKB WALLAC, Finland) were used. The result of vitamin D concentration was obtained in ng/ml, and the possible measurement error was estimated at > ± 1.5ng/ml. Statistical analysis of measurement results was performed using SAS software (mean, *p-value*, standard deviation).

### RNA isolation, miRNA library construction and NGS sequencing

Total RNA isolation from 48 lung samples was performed using the Direct-zol RNA Miniprep Kits (ZYMO Research, Orange, California) in line with the recommendations of the manufacturer. Then, the isolated genetic material was cleaned up using Monarch® RNA Cleanup Kit (New England Lab, Woburn, USA). The quality and quantity of RNA were assessed using the Tapestation 2200 (Agilent, Santa Clara, California, USA). The integrity number equivalent (RIN) scores in all RNA samples were higher than 7. The quantity of RNA was additionally evaluated by NanoDrop™ 2000/2000c Spectrophotometers (Thermo Scientific™, Foster City, California, USA). Next, the isolates were used for library preparation with the NEBNext Multiplex Small RNA Library Prep Set for Illumina (New England Lab, Woburn, USA). The quantity assessment of pooled libraries was performed using the Qubit (Ther-mo Scientific™, Foster City, California, USA), while the quality was assessed using the Tapestation 2200 (Agilent, Santa Clara, California, USA) devices. Sequencing of pooled libraries (75 bp single read) was performed on the NextSeq 550 device (Illumina, San Diego, California, USA) at the National Research Institute of Animal Production in Balice. The libraries for sequencing were prepared according to the Standard Normalization Method from NextSeq 500 and NextSeq 550 Sequencing Systems-Denature and Dilute Libraries Guide protocol. We used a 2nM starting library concentration and 10% PhiX addition.

### Statistics of the miRNA-seq results

The obtained reads were demultiplexed with bcl2fastq software (Illumina) and quality controlled with FastQC software. The reads were also subjected to adapter and length trimming (18–25 nt) with the TrimGalore package. miRDeep2 was applied to identify known and potentially novel miRNAs using default settings, Sus scrofa 11.1 reference genome, miRBase 22.1. R package and DESeq2 software were used to perform the differential expression analysis. miRNAs with *p-adjusted* < 0.05 Benjamini–Hochberg (BH) adjustment and no fold-change threshold were regarded as differentially expressed (DE). Only those miRNAs for which the number of reads per sample was greater than zero in a minimum of four samples in each group were used for further analysis. Functional analysis of differentially expressed (DE) miRNAs was conducted with DIANA-miRPath v3.0 online tool for human miRNA homologs.

### qPCR analysis

The RNA from 28 samples (7 samples/group) was reverse transcribed using a miRCURY LNA RT Kit (QIAGEN, Hilden, Germany), according to the manufacturer protocol. The following miRNAs were selected to confirm the results obtained from miRNA-seq: miR-215-5p, miR-96-5p and miR-381-3p. The selection was guided by the average number of reads and the log2FoldChange value. Real-time PCR was performed using miRCURY LNA SYBR Green PCR Kit (QIAGEN, Hilden, Germany) and miRCURY LNA miRNA PCR Assays (QIAGEN, Hilden, Germany) on a QuantStudio™ 7 Flex Real-Time PCR System (Applied Biosystems™, Waltham, Massachusetts, United States). Relative quantity data were analysed on the Thermo Fisher Cloud (Thermo Scientific). The results obtained from NGS (Next Generation Sequencing) and those obtained from qPCR analysis were compared by checking the level of Pearson correlation (r2).

The RNA isolated from the 28 samples was additionally reverse transcribed using the High-Capacity RNA-to-cDNA™ Kit (Applied Biosystems™, Waltham, Massachusetts, United States). The resulting cDNA was used to analyse changes in the expression of the *NEU1* (Neuraminidase 1) and *FUT1* (Fucosyltransferase 1) genes using *RPS29* endogenous control. These genes are targets of miRNAs altered by the vitamin D supplementation and were selected based on the results of functional analysis of miRNA-seq results. Analysis of the obtained results was conducted using the Thermo Fisher Cloud (Thermo Scientific). The level of significance of differences between groups was checked using the Mann-Whitney U and t-tests.

## Results

The 25(OH)D blood test confirmed that the applied supplementation was effective. The plasma concentration of 25(OH)D in animals from all experimental groups differed significantly (*p-value* < 0.05) from that of the control group.

Pigs supplemented with a standard dose of cholecalciferol had the lowest plasma 25(OH)D concentration (39.67 ng/ml, +/- 10.25). The mean 25(OH)D concentration in animals receiving the increased cholecalciferol dose was 24.29 ng/ml higher (63.96 ng/ml, +/- 22.68). Animals treated with calcidiol (groups 3 and 4) had significantly (*p-value* < 0.05) higher vitamin D concentrations compared to the others. Animals receiving cholecalciferol + calcidiol combinations had an average of 124.93 ng/ml (+/- 23.20), while those receiving calcidiol alone had non-significantly higher plasma 25(OH)D concentrations- 133.5 ng/ml (+/- 20.5).

### RNA assessment and miRNA-seq statistics

RNA evaluation indicated that 39 samples met the quality criteria (RIN scores > 7). The remaining 8 samples were excluded from further analysis.

The NGS of lung tissue samples proceeded correctly for 38 of 39 samples. The average number of mapped reads was 345 116 per sample. One sample was discarded due to a low number of mapped reads (8569 reads). All 38 samples were used for further analysis, including miRNA identification and differential expression analysis. The sequencing results have been deposited in the NCBI GEO database (accession number: GSE217599).

### Differential expression analysis results

Table 1. shows the miRNAs which, according to adjusted *p-value* (*q-value* < 0.05), were most significantly altered. The properties of individual miRNAs recognized so far are compiled in the table in Table S1. Nine significantly altered miRNAs were excluded from further analysis because the number of reads for these miRNAs was zero for most samples in each group.

The obtained results (Table 1.) indicate that the use of the increased dose of cholecalciferol compared to the standard dose (1 vs. 2) caused a significant change (*q-value* = 0,004) in the miR-215-5p expression only (log2FoldChange = 2,652). In turn, supplementation with the increased dose of the cholecalciferol + calcidiol combination (1 vs. 3) resulted in the altered expression of 13 miRNAs, including 7 upregulated and 6 downregulated. In contrast, supplementation with the standard dose of calcidiol instead of cholecalciferol (1 vs. 4) showed no effect on the miRNA profile.

Subsequent analysis showed that the increased dose of cholecalciferol + calcidiol compared to the increased dose of cholecalciferol (2 vs. 3) showed a change in the expression of 12 miRNAs (Table 1.). Among them, 9 miRNAs were upregulated and 3 were downregulated. The comparison of the standard dose of calcidiol with the increased dose of cholecalciferol + calcidiol (4 vs. 3) showed 17 altered miRNAs. However, 11 of these miRNAs are repeated in the results of the other comparisons, and the direction of their changes is the same. Only 6 of the altered miRNAs were specific for the 4 vs. 3 comparison, of which 5 miRNAs were downregulated and 1 was upregulated.

**Table 1:** The miRNAs altered by different doses and forms of vitamin D supplementation (*q-value* < 0.05). The level of change is presented in log2FoldChange (log2FC).

2 vs. 1		3 vs. 1		3 vs. 2		3 vs. 4	
miR name	log2FC	miR name	log2FC	miR name	log2FC	miR name	log2FC
miR-215-5p	2,652	miR-574-3p	1,663	miR-4334-3p	2,727	miR-885-5p	1,944
		miR-193a-5p	1,249	miR-205-5p	2,69	miR-574-3p	1,503
		miR-145-5p	1,101	miR-885-5p	2,337	miR-193a-5p	1,329
		miR-150-5p	1,015	miR-125b-5p	2,157	miR-150-5p	0,938
		miR-181a-5p	0,756	miR-574-3p	2,047	miR-181a-5p	0,72
		miR-191-5p	0,674	miR-133a-3p	1,691	miR-676-3p	0,604
		miR-181b-5p	0,519	miR-145-5p	1,311	miR-181b-5p	0,505
		miR-340-5p	-0,86	miR-193a-5p	1,145	miR-21-5p	-0,869
		miR-450a-5p	-0,885	miR-150-5p	0,887	miR-340-5p	-0,917
		miR-101-3p	-1,228	miR-148b-3p	-0,984	miR-218-5p	-0,923
		miR-96-5p	-1,653	miR-151a-3p	-1,695	miR-450a-5p	-0,963
		miR-182-5p	-2,117	miR-215-5p	-2,313	miR-148b-3p	-0,972
		miR-381-3p	-2,599			miR-299-3p	-1,176
						miR-183-5p	-1,203
						miR-96-5p	-1,36
						miR-101-3p	-1,504
						miR-381-3p	-2,504

### qPCR analysis

One of the reverse-transcribed miRNA samples was excluded at a further stage of analysis due to a significant concentration deviation from the other cDNA samples. The qPCR validation performed for 27 samples partially confirmed the NGS results (Table S2.). The Pearson correlation (r2) coefficient for the miR-215-5p was > 0.63 (*p-value* = 0.0004), for miR-381-3p was > 0.60 (*p-value* = 0.0008), and for the miR-96-5p was > 0.58 (*p-value* = 0.0015).

In turn, analysis of *NEU1* and *FUT1* gene expression carried out on 28 samples showed no significant differences between groups (Figure S1.). The *FUT1* expression results were not characterized by a normal distribution, because of this we performed a non-parametric analysis for this data.

### Functional analysis

For the functional analysis we used the differentially expressed miRNAs from 1 vs. 3, 2 vs. 3 and 4 vs. 3. The analysis was conducted using DIANA software (‘pathways union’ option) based on a human database. The results indicated that the increased dose of cholecalciferol + calcidiol significantly (*p-value* < 0.05) affected 15 KEGG pathways compared to the standard cholecalciferol supplementation (Table 2.). Among them, there were 5 disease-related pathways, and 4 were connected to cancers (cancer overview, glioma). On the other hand, the pathways from the organismal systems class are related to the endocrine system (2 pathways), nervous system (1 pathway), excretory system (1 pathway) as well as development and regeneration (1 pathway). The remaining altered pathways were associated with signal transduction, cellular community and xenobiotics biodegradation and metabolism.

**Table 2:** KEGG pathways that were stimulated by the combined supplementation of cholecalciferol + calcidiol in comparison to cholecalciferol at standard doses in the lungs of finishing pigs

KEGG pathway	*p-value*	KEGG class
Estrogen signalling pathway	8.7e-09	Organismal Systems; Endocrine system
TGF-β signalling pathway	2.7e-08	Environmental Information Processing; Signal Transduction
Signalling pathways regulating pluripotency of stem cells	1.8e-05	Cellular Processes; Cellular community - eukaryotes
Phosphatidylinositol signalling system	2.5e-05	Environmental Information Processing; Signal Transduction
Proteoglycans in cancer	6.4e-05	Human Diseases; Cancer: Overview
Amphetamine addiction	0.0001	Human Diseases; Substance dependence
Metabolism of xenobiotics by cytochrome P450	0.004	Metabolism; Xenobiotics biodegradation and metabolism
Transcriptional Misregulation in Cancer	0.005	Human Diseases; Cancer: Overview
Adherens junction	0.012	Cellular Processes; Cellular community - eukaryotes
Axon guidance	0.028	Organismal Systems; Development and regeneration
Vasopressin-regulated water reabsorption	0.033	Organismal Systems; Excretory system
Glioma	0.037	Human Diseases; Cancer: specific types
GABAergic synapse	0.039	Organismal Systems; Nervous system
Oxytocin signalling pathway	0.040	Organismal Systems; Endocrine system
Pathways in cancer	0.044	Human Diseases; Cancer: Overview

**Fig. 2:**
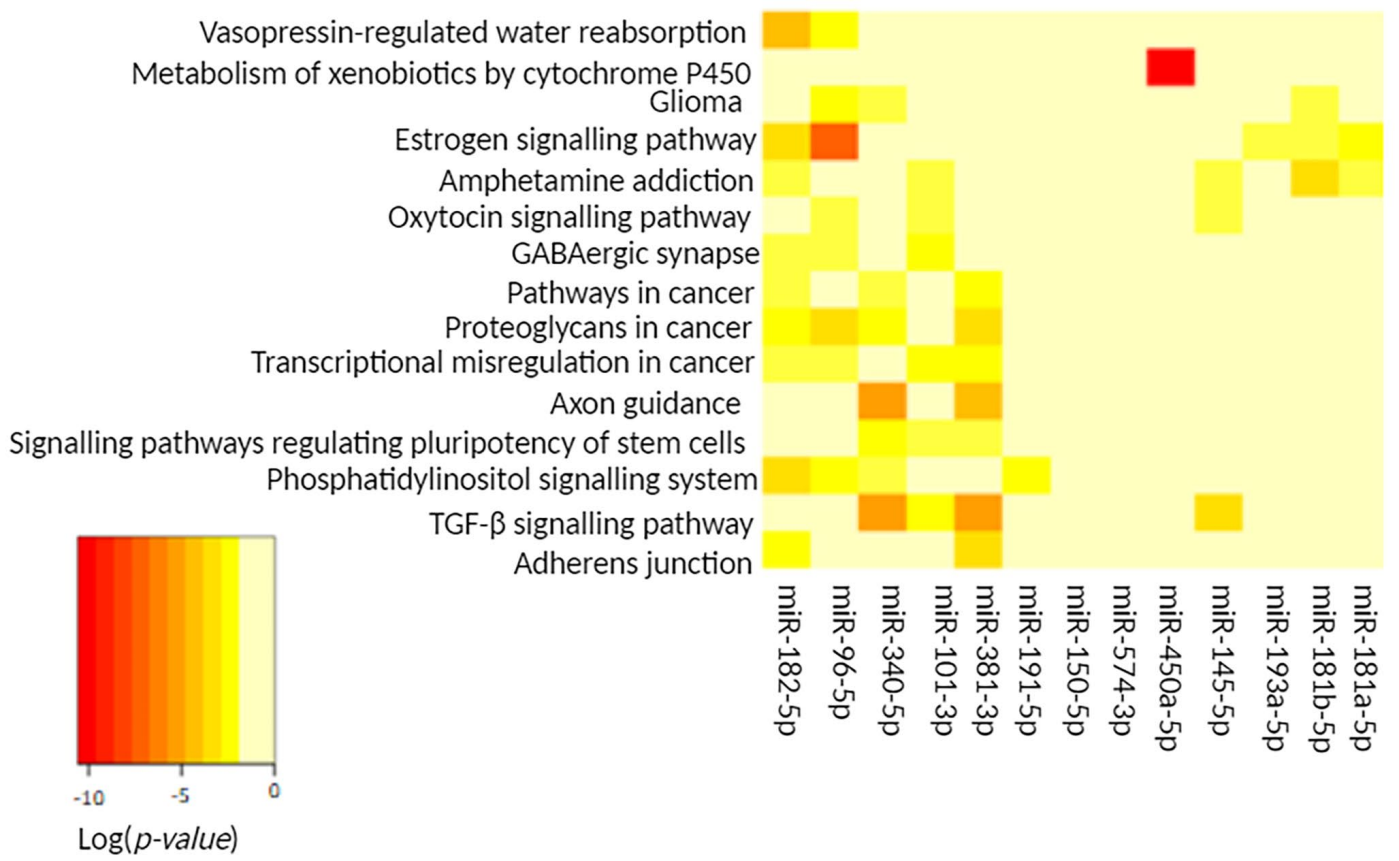
The heat map shows the miRNAs altered by the increased dose of cholecalciferol + calcidiol (1 vs. 3), the pathways affected, and the clusters formed by each of the altered miRNAs and pathways

Supplementation of the increased dose of cholecalciferol + calcidiol had the most significant effect on the estrogen signalling pathway (*p-value* = 8.74E-09), TGF-β signalling pathway (*p-value* = 2.69E-08), signalling pathways regulating pluripotency of stem cells (*p-value* = 1.80E-05), phosphatidylinositol signalling system (*p-value* = 2.45E-05) and proteoglycans in cancer (*p-value* = 6.42E-05). Figure 2 presents miRNAs and pathways clusters, as well as the number and significance of each miRNA’s contribution to the regulation of the altered pathway. The heatmap shows that 5 of the 13 differentially expressed miRNAs are associated with the estrogen signalling pathway. Similarly, in the case of amphetamine addiction, 5 of the changed miRNAs are related to this substance-dependence disease. In contrast, only 1 of the altered miRNAs (miR-450a-5p) is associated with the pathway of metabolism of xenobiotics by cytochrome P450, but the significance of miR-450a-5p for this pathway is particularly high.

Among the altered miRNAs engaged in the highest number of pathways are miR-182-5p (9 pathways), miR-96-5p (8 pathways), miR-340-5p (7 pathways), miR-381-3p (7 pathways) and miR-101-3p (6 pathways).

**Fig. 3:**
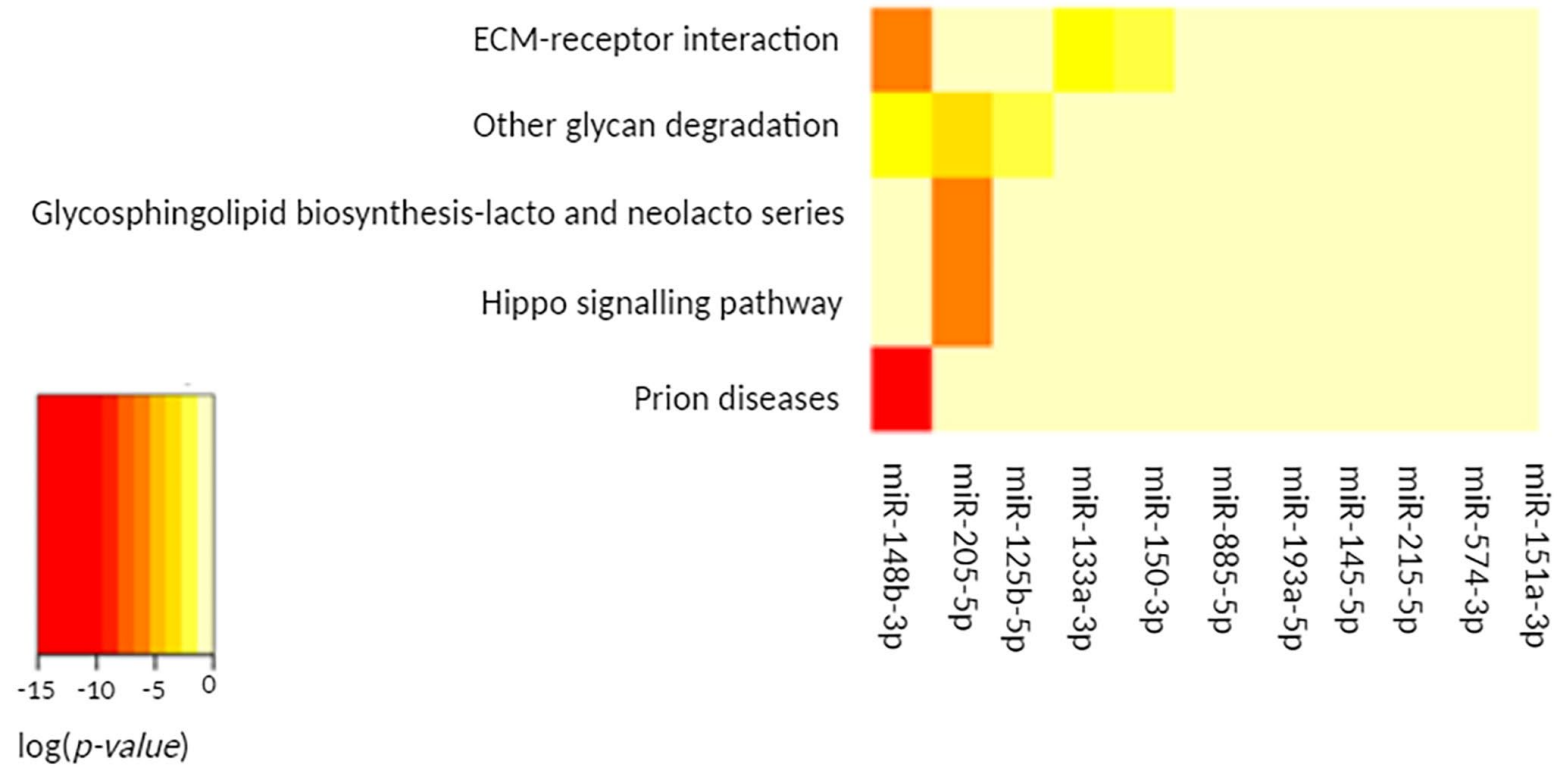
The heat map shows the miRNAs altered by replacing a part of cholecalciferol with calcidiol at the increased dose (2 vs. 3), the pathways affected, and the clusters formed by each altered miRNA and pathway

The partial replacement of cholecalciferol with calcidiol at the increased dose (2 vs. 3) had a significant effect on 5 pathways (*p-value < 0.05*) (Table 3.). The strongest effect was observed on the prion disease pathway (*p-value = < 1e-325*). This effect was caused by a change in only one miRNA expression level - miR-148b-3p (Fig. 3.). Interestingly, the results also indicate activation of two pathways related to glycan biosynthesis and metabolism; glycosphingolipid biosynthesis - lacto and neolacto series (*p-value* = 0.022) and other glycan degradation (*p-value* = 0.049). Simultaneously, the ECM-receptor interaction pathway, which can also bind to glycans, was also significantly activated (*p-value* = 0.0003).

**Table 3:** KEGG pathways that were stimulated by the combined supplementation of cholecalciferol + calcidiol in comparison to cholecalciferol at the increased dose (2 vs. 3) of finishing pigs

KEGG pathway	*p-value*	KEGG class
Prion diseases	< 1e-325	Human Diseases; Neurodegenerative disease
ECM-receptor interaction	0.0002	Environmental Information Processing; Signalling molecules and interaction
Hippo signalling pathway	0.020	Environmental Information Processing; Signal Transduction
Glycosphingolipid biosynthesis-lacto and neolacto series	0.021	Metabolism; Glycan biosynthesis and metabolism
Other glycan degradation	0.048	Metabolism; Glycan biosynthesis and metabolism

Only 5 of the 11 altered miRNAs had a significant impact on the listed pathways (Fig. 3.). Admittedly, the arrangement of clusters presents an intermediate connection between all the miRNAs; however, it is miR-148b-3p and miR-205-5p that are most relevant to the biological pathways presented in Table 3.

The functional analysis led to the finding that the use of the cholecalciferol + calcidiol combination compared to the standard calcidiol dose (4 vs. 3) significantly affects 13 biological pathways (Table 4.). Most of these (10 pathways) are identical in the comparison of the standard cholecalciferol dose with the combination (1 vs. 3) (Figure S2.). Moreover, two of the remaining pathways are specific for the comparison of the increased cholecalciferol dose with the combination (2 vs. 3). The most significant difference between the results of these comparisons is the change in miR-148 expression. This miRNA is highly significant for the prion diseases pathway, which is also the most significantly altered pathway in the results of the 2 vs. 3 and 4 vs. 3 comparisons.

**Table 4:** KEGG pathways that were stimulated by the combined supplementation of cholecalciferol + calcidiol in comparison to calcidiol (4 vs. 3) of finishing pigs

KEGG pathway	*p-value*	KEGG class
Prion diseases	< 1e-325	Human Diseases; Neurodegenerative disease
Signalling pathways regulating pluripotency of stem cells	1.1e-07	Cellular Processes; Cellular community - eukaryotes
Estrogen signalling pathway	9.9e-06	Organismal Systems; Endocrine system
TGF-β signalling pathway	1.7e-05	Environmental Information Processing; Signal Transduction
Proteoglycans in cancer	9.3e-05	Human Diseases; Cancer: Overview
Amphetamine addiction	0.0003	Human Diseases; Substance dependence
Phosphatidylinositol signalling system	0.002	Environmental Information Processing; Signal Transduction
GABAergic synapse	0.007	Organismal Systems; Nervous system
Morphine addiction	0.026	Human Diseases; Substance dependence
Glioma	0.029	Human Diseases; Cancer: specific types
Hippo signalling pathway	0.030	Environmental Information Processing; Signal Transduction
Pathways in cancer	0.048	Human Diseases; Cancer: Overview
Metabolism of xenobiotics by cytochrome P450	0.049	Metabolism; Xenobiotics biodegradation and metabolism

**Fig. 4:**
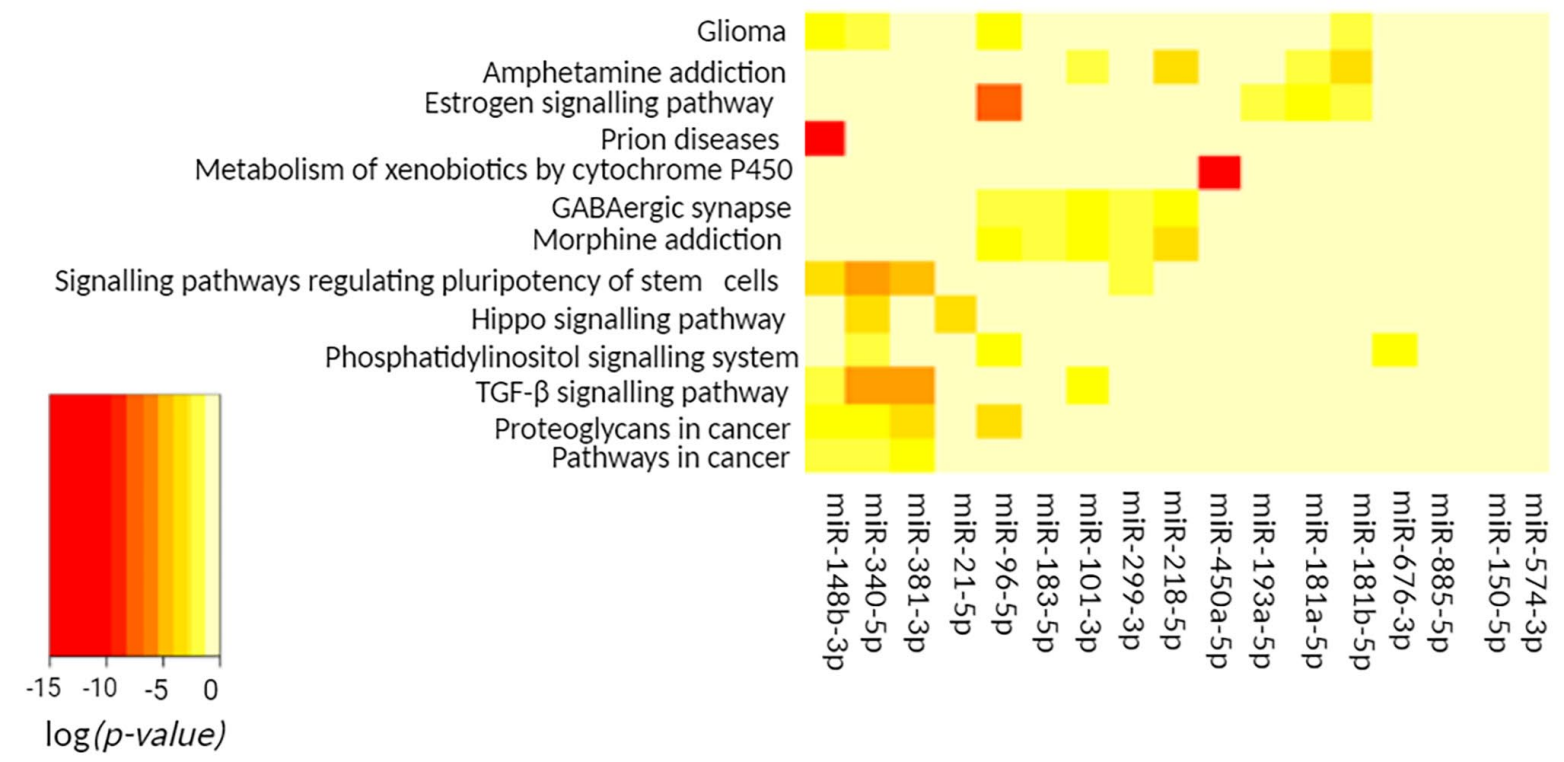
The heatmap shows the miRNAs altered by the increased dose of cholecalciferol + calcidiol compared to the standard dose of calcidiol (4 vs. 3), the pathways affected, and the clusters formed by each altered miRNA and pathway

The heatmap (Fig. 4.) illustrating the effect of supplementation with the standard dose of calcidiol compared to the increased dose of cholecalciferol + calcidiol (3 vs. 4) shows similarities to the heatmap 1 vs. 3 comparison (Fig. 2.). This underlies the similarity of miRNA profiles of samples with the standard doses of cholecalciferol or calcidiol.

## Discussion

In this study, we performed pairwise comparisons of different doses and forms of vitamin D to elucidate its effect on miRNA expression levels in healthy swine lungs. Previous studies indicate a significant association of vitamin D with the development of lung diseases, and the use of alternative forms of vitamin D such as calcidiol to treat lung diseases is of growing interest [16]. However, this is the first study describing the effects of calcidiol and choleclaciferol + calcidiol supplementation on the lung tissue miRNAome of any species.

We found that supplementation with the increased dose of the combination of cholecalciferol and calcidiol (group 3) in comparison to the other treatments induced the greatest changes in the miRNA profile of the lungs. Importantly, many of the miRNAs presented in our results appear to be typical for the lung (miR-148b, miR-101, miR-21, miR-145, miR-181, miR-191, miR-215), and they also have been mentioned in the results of other studies on this tissue [17, 18].

Many studies on the effects of vitamin D supplementation conducted to date indicate that increasing plasma 25(OH)D concentration has an immunomodulatory effect on lung tissue [19, 20]. However, the results presented here shed new light on these findings. We observed that even a significant increase in 25(OH)D concentration induced by calcidiol supplementation (group 4) did not remarkably affect the lung miRNA profile. Moreover, an almost equally high increase in plasma 25(OH)D concentration induced by supplementation with the cholecalciferol + calcidiol combination caused significant changes in the lung tissue transcriptome. This may suggest that simple increasing plasma 25(OH)D concentration may be insufficient to cause a change at the level of gene regulation and metabolic transformation of inactive vitamin D in the liver are necessary to achieve this.

### The effect of increased dose of cholecalciferol

The first stage of our analysis was to determine how increasing the dose of commonly used cholecalciferol affects the miRNA profile of healthy lungs. We found that increasing the dose of cholecalciferol from 2000 IU to 3000 IU had little effect regarding the number of altered miRNAs. MiR-215, the only differentially expressed miRNA, is involved in several basic processes such as cell and tissue development, cell survival and migration, cell cycle and proliferation, as well as cellular metabolism [21]. Due to its crucial functions, the dysregulation of miR-215 expression has been implicated in the pathogenesis of many diseases. Changes in miR-215 expression in different types of cancer have been researched extensively. For some types of tumours, this miRNA is a suppressor, for others - an oncogene [21]. Moreover, one recent finding demonstrated a strong link between miR-215 and the development of pulmonary fibrosis [22]. The study also indicated that miR-215 was upregulated in herbicide-induced pulmonary fibrosis (in vitro and in vivo) causing the activation of the TGF-β pathway by inhibiting the expression of the *BMPR2* target gene. The research concluded that reducing miR-215 expression decreased the progression of irreversible pulmonary fibrosis [22]. In our experiment, the increased dose of cholecalciferol (1 vs. 2) showed a significant upregulation of the expression of miR-215 (log2FoldChange = 2, 65). In contrast, subsequent analyses indicated that the use of the same dose of the cholecalciferol + calcidiol combination (1 vs. 3) did not affect this miR-215 Therefore, further research is necessary to clearly determine how vitamin D supplementation is related to the dysregulation of this miRNA.

### The effect of replacing cholecalciferol with calcidiol

The second step of our analysis was to test the effect of calcidiol vs. cholecalciferol on the miRNA profile. Calcidiol is significantly more effective in raising the concentration of 25(OH)D in plasma compared to cholecalciferol. Therefore, we wanted to investigate if this higher efficacy may also be followed by the changes related to gene expression regulation. Surprisingly, we found that the miRNA lung profile of swine supplemented with the same dose (2000 IU/kg) of calcidiol and cholecalciferol did not differ significantly. In vitro studies demonstrated that 25(OH) vitamin D and 1,25(OH)_2_ vitamin D have overlapping effects on gene expression but each of this metabolite also display partially independent gene transcriptional effects [23]. Nevertheless, the concentrations of vitamin D metabolites used in in vitro studies are much higher than that observed in living organisms, thus 1,25(OH)2 is the sole active metabolite under physiologic conditions. The results of our studies suggest that increasing the level of 25(OH) vitamin D by providing calcidiol, bypassing enzymatic transformations in the liver, has a negligible effect on the miRNA expression in the lungs. Perhaps greater differences in the action of two forms of vitamin D could be observed in the liver or kidneys - places where the main steps of vitamin D metabolism occur.

### The effect of replacing cholecalciferol with the cholecalciferol and calcidiol combination

In contrast, the use of the increased dose of cholecalciferol + calcidiol showed several changes in miRNA expression compared to supplementation with cholecalciferol (1 vs. 3, 2 vs. 3) or calcidiol alone (4 vs. 3). This result was also surprising as one could rather expect a linear increase in the number of miRNAs changed with increasing 25(OH) vitamin D plasma concentration. Although there are several experiments describing the effects of replacing calciferol with calcidiol in pigs [24] we could not find any reports on the effect of the combination of these two substances. Molecular mechanisms that regulate vitamin D metabolism and activation are very complicated and include among others the availability of Ca ions. There is also a negative feedback loop by which 1,25(OH)_2_ vitamin D inhibits the expression of 1α-hydroxylase (CYP27B1), preventing excessive 1,25 (OH)_2_ vitamin D concentration. Thus the possible explanation of the highest effect of calciferol/calcidiol combination on miRNAs expression in our study could be that there is some optimal concentration of different vitamin D metabolites that triggers the changes at the miRNA level.

The effect of the cholecalciferol + calcidiol combination compared to cholecalciferol alone (3 vs. 1 and 2 vs. 3) provides interesting implications based on the changes in the expression of 4 common findings: miR-150, miR-193, miR-145, miR-574.

First- miR-150 - regulates the expression level of TLR2, a receptor involved in the primary mechanism of the immune response that protects against bacterial and viral infections [25]. Presumably, miR-150 may also play an important role in virulence through the regulation of transcription factor- c-Myb [26]. Moreover, Zheng et al. found, that miR-150 is downregulated in macrophages in tuberculosis (TB) patients [26]. On the other hand, miR-193, identified by us as upregulated by cholecalciferol + calcidiol, is overexpressed in macrophages in TB patients [26].

Nevertheless, due to the biological function of miR-193a (modulation of cell proliferation), it is worth taking a closer look at the changes in the expression level of this miRNA. According to Khordadmehr et al., miR-193a can be a valuable tool for lung cancer prognosis and diagnosis [27]. Furthermore, Yu et al. indicated that miR-193a overexpression inhibits non-small cell lung cancer (NSCLC) cell migration, invasion and epithelial-mesenchymal transition, and lung metastasis formation in vivo [28]. Thus, it may suggest that miR-193a acts as a tumour suppressor and cholecalciferol + calcidiol supplementation has a valuable effect on lung tissue in this regard. This is also confirmed by the change in expression of another tumour suppressor - miR-145 - which was also significantly upregulated under the cholecalciferol + calcidiol combination [29]. A study by Li et al. proves that miR-145 shows high expression in healthy lung tissue, while in NSCLC cells its expression is strongly reduced [30]. Moreover, it was observed that miR-145 may regulate pro-inflammatory and anti-inflammatory effects after intracellular bacterial infection in epithelial cells [31]. It has been found that downregulation of miR-145 expression inhibits eosinophilic inflammation, excessive mucus secretion, T(H)2 cytokine production and airway hyperresponsiveness [32]. Additionally, studies on the effects of exposure to tobacco smoke, conducted on rats, have shown that air pollution causes significant downregulation of miR-145 [33]. This finding is also supported by a study which used human tissues [34]. This study suggested that increased miR-145 expression alleviated apoptosis and inflammatory response by regulating apoptotic signalling mediated (*p53*) and pre-inflammatory factors (*TNF-α, IL-6, IL-8*) in bronchial epithelial cells [34]. Considering these results, the increase in miR-145 expression under the influence of cholecalciferol + calcidiol supplementation appears to be beneficial for mammals including those constantly exposed to air pollution.

On the other hand, we can reach different conclusions by analyzing the change in miR-574 expression (upregulation). The results of research conducted on a group of healthy individuals exposed to feed dust suggest that increased expression of miR-574 is a good predictor of such exposure [35]. Moreover, oncology studies also point to negative effects associated with increased miR-574 expression. It seems to be well-confirmed that *TLR9* signalling increases the expression of miR-574 in human lung cancer cells. A meticulous in vitro and in vivo study indicated that Ches1-mediated upregulation of miR-574 enhanced tumour progression [36]. However, the in-silico study we performed showed that miR-574 is not significantly associated with any of the four cancer-related pathways. Therefore, the results of a recent study by Wei et al. seem to be more interesting [37]. These researchers discovered a mechanism of circ_0001498 action that stimulates sepsis-induced acute lung failure syndrome. Circular RNAs (circRNAs) are stable and conserved RNAs that serve as miRNA sponges affecting gene regulation. The circ_0001498 is a miR-574 sponge, and *SOX6* is a target gene of miR-574. Wei et al. found that overexpression of circ_0001498 mediated by miR-574 upregulates *SOX6*. In contrast, overexpression of miR-574 had the opposite effect (downregulation of *SOX6*), resulting in attenuation of cell damage [37]. The effect of the increased miR-574 expression on attenuating sepsis-induced lung injury was also confirmed in another study [38]. However, their effect was presumably associated with a reduction in *C3* (inflammatory transmitter regulated by miR-574) levels and a decrease in sepsis-induced endoplasmic reticulum stroma [38]. With the results cited, it can be concluded that upregulation of miR-574 induced by cholecalciferol + calcidiol supplementation may show a protective effect on lung tissue cells, both by downregulation of *SOX6* and *CD3* levels.

Among the miRNAs altered by the cholecalciferol + calcidiol combination, it is also worth mentioning about downregulation of miR-148b. Results of an experiment performed by Pacholewska et al. on healthy and asthmatic horses showed significantly higher expression (*p-value* = 0,043) of this miRNA in the blood of asthmatic horses [39]. The association of miR-148 with asthma was also pointed out by researchers experimenting on human cell lines. They suggested that miR-148b may contribute to the risk of asthma by regulating the HLA-G-a molecule with immunomodulatory properties [40]. Moreover, asthmatic horses have also been characterized by significantly (*p-value* = 0,05) higher expression of miR-215 [39]. Interestingly, our study showed a significant decrease in miR-215 expression levels under cholecalciferol + calcidiol supplementation (2 vs. 3) and a significant rise in the expression of this miRNA caused by the increased dose of cholecalciferol alone (1 vs. 2).

The opposing functions of individual miRNAs presented above illustrate the complexity of the mechanisms in which they participate. Moreover, the vast majority of miRNA profile analyzes concern patients. For these reasons, a clear assessment of the effect of changes in the expression of individual miRNAs requires additional research, including mRNA analysis.

Nevertheless, our results indicate that the cholecalciferol + calcidiol combination can regulate the cancer formation processes and functioning of the immune system by influencing the mentioned miR-150, miR-193, miR-145 and miR-574.

### Functional analysis of differentially expressed miRNAs after cholecalciferol + calcidiol supplementation

In the functional analysis, we focused on miRNAs that were differentially expressed after the increased dose of cholecalciferol + calcidiol compared to a standard dose of cholecalciferol or calcidiol (1 vs. 3 and 4 vs. 3). There were 10 pathways common for these comparisons. Among them, the most significantly altered were signalling pathways regulating pluripotency of stem cells, estrogen signalling pathway, TGF-β signalling pathway and proteoglycans in cancer (Table 2. and Table 4.). Most of the identified pathways are associated with tumorigenesis, and only a few are linked to other functions attributed to vitamin D (e.g. Metabolism of xenobiotics by cytochrome P450). The reason for this may be that oncology research accounts for the vast majority of all miRNA research.

The signalling pathway regulating stem cell pluripotency is, among others, influenced by miR-340, miR-381 and miR-148. The results of our study indicate that replacing standard cholecalciferol supplementation with the increased dose of the combination of different forms of vitamin D has a significant effect on the expression of miR-340 and miR-381. Additionally, the combination use compared to the standard dose of calcidiol also alters the expression of miR-148. One of the targets of miR-340 is *Sox2*, and for miR-148 it is the *Klf4* gene. Both genes belong to a subsatellite transcriptional network that activates the reprogramming of somatic cells back to a pluripotent state. Moreover, both genes are thought to be crucial to the mechanisms of cancer cell proliferation [41]. Another pathway of interest in this regard and highly activated under increased cholecalciferol + calcidiol supplementation was the TGF- β signalling pathway. Our functional analysis showed that this pathway was regulated by changes in the expression of miR-381, miR-101, miR-148 and miR-340. *SMAD* genes (*SMAD2, SMAD9, SMAD5, SMAD4*) targeted by these miRNAs modulate both proliferation and apoptosis as well as cell differentiation and migration [42]. One research showed that vitamin D, through its effect on the TGF- β pathway, prevents cancer cell-induced apoptosis of inflammatory cells [43]. Moreover, Moz et al., demonstrated that vitamin D can reduce the depletion of peripheral blood mononuclear cells (PBMCs) induced by cancer cells. Furthermore, this research also indicated that vitamin D inhibits tumour cell-induced release of tumour necrosis factor-alpha (TNF-α) and reduces intracellular transforming growth factor beta (TGF-β) levels [43].

Another activated path - estrogen signalling - is closely linked to the regulation of gene expression via, e.g., miRNAs [44]. We observed that the significance of the estrogen signalling pathway was determined particularly by the change in expression of miR-96, followed by miR-181a, miR-181b and miR-193a (Figs. 2 and 3.). Action of calcidiol related to the regulation of estrogen synthesis and signalling may determine the anticancer effect of this vitamin D metabolite [45]. Furthermore, calcidiol has been found to inhibit *COX-2* expression and increase *15-PGDH* expression, thereby reducing inflammatory mediator expression (prostaglandins). Thus, the putative inhibition of estrogen synthesis and signalling by calcidiol, as well as its anti-inflammatory properties, may play an important role in the prevention and treatment of cancer [45].

Also, the last of the above-mentioned pathways - proteoglycans in cancer - shows a very wide range of activities with tumour cells. Proteoglycans contribute to proliferation, adhesion, angiogenesis and metastasis, thus influencing the biology of various types of cancer significantly [46–48]. One well-known proteoglycan is hyaluronan (HA), which acts with *CD44* to enhance the growth and migration of tumour cells. The significance of the effect on this pathway was determined by the cholecalciferol + calcidiol-induced changes in miR-381, miR-96, miR-148 as well as miR-340 expression levels. Remarkably, the use of the increased dose of the cholecalciferol + calcidiol combination compared to the same dose of cholecalciferol alone could also activate glycosphingolipid biosynthesis - lacto and neolacto series - and other glycan degradation pathways belonging to the class of glycan biosynthesis and metabolism (Table 3.). A significant effect on the former pathway is related to the change in the expression of miR-205. The latter-another glycan degradation pathway is additionally regulated by changes in the expression of miR-125 and miR-148 (Fig. 3.). Interestingly, the potential link between vitamin D and proteoglycans was also highlighted in another experiment. A study by D’arrigo et al. showed that activation of the vitamin D receptor (VDR) through applications of paricalcitol raises the level of one of the proteoglycans – thrombomodulin (TM), which in turn, improves endothelial function [49]. All this information taken together, suggests that increased cholecalciferol + calcidiol supplementation may modulate the cell microenvironment by affecting glycan synthesis and metabolism.

Based on these findings, we decided to test the potential of vitamin D in regulating glycoprotein metabolism. For this purpose, we selected two genes (*NEU1* and *FUT1*) that are involved in glycoprotein metabolism and which are the targets of miRNAs significantly altered under cholecalciferol + calcidiol supplementation (miR-125b, miR-205). The first of the selected genes- *NEU1* encodes neuraminidase1. This enzyme plays an important role in various biological processes such as cell recognition, adhesion, cell signalling as well as degradation of glycoprotein molecules. The second gene - *FUT1* encodes an enzyme called fucosyltransferase 1, which is involved in the process of adding fucosyl sugars to glycans on the surface of cells. However, the analysis we performed did not show statistically significant differences in the expression of these genes, suggesting different ways of action of differentially expressed miRNAs.

## Conclusions

Our results indicate that the increased dose of cholecalciferol + calcidiol causes many significant changes in the miRNA profile compared to cholecalciferol-only supplementation in the lungs of finishing pigs. Among the altered miRNAs, we can distinguish those that appeared in the results of several comparisons - miR-150, miR-193, miR-145, miR-574, and those that turned out to be the most significant from the point of view of functional analysis- miR-340, miR-381, miR-148 and miR-96. Simultaneously, we showed that the total exchange of standard cholecalciferol for calcidiol does not cause significant changes in the miRNA profile. The results of the functional analysis suggest that the cholecalciferol + calcidiol combination may affect tumorigenesis processes through, inter alia, modulation of the TGF- β pathway and pathways related to metabolism and glycan synthesis.

The miRNAs we have identified have the potential to affect dozens of genes. Therefore, the results presented above provide valuable information and a stimulus for further research including mRNA profiling results, DNA methylation analysis and clinical trials to fully report on the global molecular effects of dietary use of different doses and forms of vitamin D.

## Electronic supplementary material

Below is the link to the electronic supplementary material.

**Supplementary Material 1: Figure S1.** Mean RQ (relative quantification) for NEU1 and FUT1 genes in lung tissue in each dietary group

**Supplementary Material 2: Figure S2.** Biological pathways altered under cholecalciferol+calcidiol combination compared to standard dose of cholecalciferol (1 vs 3) and calcidiol (4 vs 3)

**Supplementary Material 3: Table S1.** List of functions of individual miRNAs altered by calcidiol and cholecalciferol+calcidiol supplementation

**Supplementary Material 4: Table S2.** Results of qPCR validation of mRNA sequencing data of pigs supplemented with different doses and forms of vitamin D

## Data Availability

The sequencing results have been deposited in the NCBI GEO database (accession number: GSE217599).
