# Supplementary material for: Changes in miRNA expression in the lungs of pigs supplemented with different levels and forms of vitamin D
Source: Mol Biol Rep. 2023 Dec 12;51(1):8. doi: 10.1007/s11033-023-08940-1 (PMC10716066; doi:10.1007/s11033-023-08940-1)
Supplement: Supplementary file 1 — Supplementary Material 1: Figure S1. Mean RQ (relative quantification) for NEU1 and FUT1 genes in lung tissue in each dietary group [file 11033_2023_8940_MOESM1_ESM.docx]

**Figure S1.** Mean RQ (relative quantification) for *NEU1* and *FUT1* genes in lung tissue in each dietary group.
