# Supplementary material for: Changes in miRNA expression in the lungs of pigs supplemented with different levels and forms of vitamin D
Source: Mol Biol Rep. 2023 Dec 12;51(1):8. doi: 10.1007/s11033-023-08940-1 (PMC10716066; doi:10.1007/s11033-023-08940-1)
Supplement: Supplementary file 2 — Supplementary Material 2: Figure S2. Biological pathways altered under cholecalciferol+calcidiol combination compared to standard dose of cholecalciferol (1 vs 3) and calcidiol (4 vs 3) [file 11033_2023_8940_MOESM2_ESM.docx]

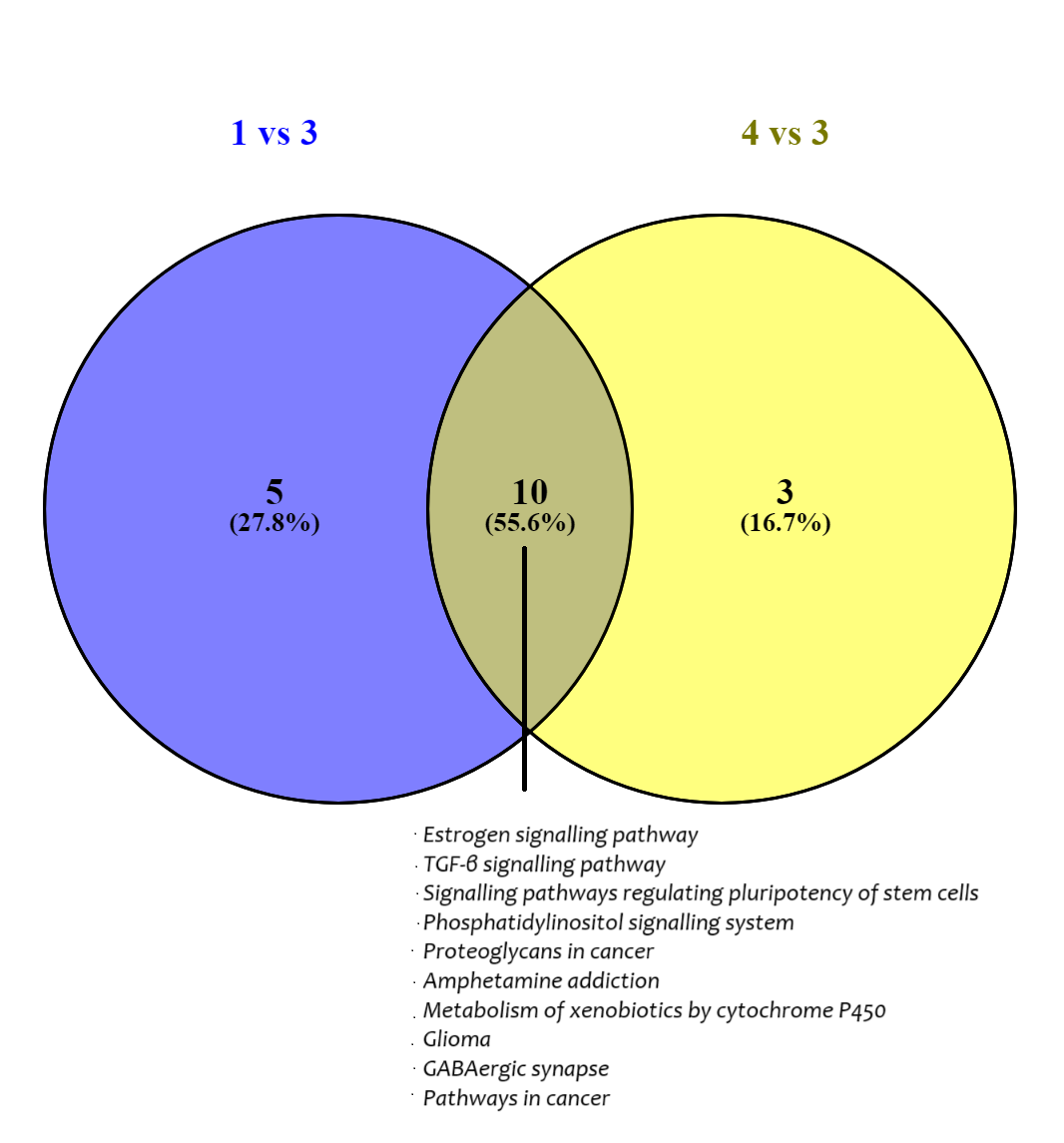


**Figure S2.** Biological pathways altered under cholecalciferol+calcidiol combination compared to standard dose of cholecalciferol (1 vs 3) and calcidiol (4 vs 3).
