# Supplementary material for: Changes in miRNA expression in the lungs of pigs supplemented with different levels and forms of vitamin D
Source: Mol Biol Rep. 2023 Dec 12;51(1):8. doi: 10.1007/s11033-023-08940-1 (PMC10716066; doi:10.1007/s11033-023-08940-1)
Supplement: Supplementary file 3 — Supplementary Material 3: Table S1. List of functions of individual miRNAs altered by calcidiol and cholecalciferol+calcidiol supplementation [file 11033_2023_8940_MOESM3_ESM.docx]

**Table S1.** List of functions of individual miRNAs altered by calcidiol and cholecalciferol+calcidiol supplementation.

| **Name** | **Function** | **Name** | **Function** |
| --- | --- | --- | --- |
| **miR-21** | blood vessel development | **miR-181a** | cell migration |
|  | cytokine production |  | TGF-β receptor signaling pathway |
|  | TGF-β receptor signaling pathway |  | iL-1α, iL-6 and iL-8 production |
|  | iL-12 production |  | tumor necrosis factor production |
|  | mitotic cell cycle | **miR-181b** | signal transduction |
|  | inflammatory response |  | mitochondrial membrane potential |
|  | vascular endothelial growth factor signaling pathway |  | innate immune response |
|  | blood vessel endothelial cell proliferation |  | inflammatory response |
|  | retinal cell apoptotic process |  | cell migration |
| **miR-96** | high-density lipoprotein particle clearance |  | gene silencing |
|  | gene silencing |  | nitric oxide biosynthetic process |
|  | cholesterol biosynthetic process |  | endothelial cell differentiation |
|  | atty acid biosynthetic process |  | angiogenesis |
|  | cellular response to cholesterol | **miR-182** | cytokine production |
|  | vascular associated smooth muscle cell proliferation |  | cell migration |
|  | SREBP signaling pathway |  | gene silencing |
| **miR-101** | protein ubiquitination |  | cholesterol homeostasis and cholesterol biosynthetic process |
|  | iL-1α and iL-6 production |  | fatty acid biosynthetic process |
|  | tumor necrosis factor production |  | lipoprotein lipase activity |
|  | DNA-binding transcription factor activity |  | cellular response to cholesterol |
|  | blood vessel endothelial cell migration |  | non-canonical NF-kappaB signal transduction |
|  | cell adhesion molecule production |  | histone deacetylase activity |
| **miR-125b** | keratinocyte proliferation |  | epithelial cell apoptotic process |
|  | angiogenesis |  | vascular associated smooth muscle cell proliferation |
|  | BMP signaling pathway |  | vascular associated smooth muscle cell migration |
|  | tumor necrosis factor production |  | vascular associated smooth muscle cell dedifferentiation |
|  | gene silencing | **miR-183** | cell-substrate adhesion, |
|  | osteoblast differentiation |  | phagocytosis |
| **miR-133a** | cell population proliferation | **miR-191** | gene silencing |
|  | cell fate commitment | **miR-193a** | angiogenesis |
|  | cardiac muscle hypertrophy |  | blood vessel endothelial cell migration |
|  | cardiac muscle cell apoptotic process |  | vascular endothelial cell proliferation |
|  | myotube differentiation | **miR-205** | endothelial cell proliferation |
|  | cardiac muscle contraction |  | angiogenesis |
|  | low-density lipoprotein particle clearance |  | cell migration |
|  | cell migration |  | osteoblast differentiation |
|  | IP3-sensitive calcium-release channel activity |  | receptor-mediated endocytosis |
|  | transporter activity |  | SMAD protein signal transduction |
|  | gene silencing |  | ERK1 and ERK2 cascade |
|  | mRNA base-pairing translational repressor activity |  | cell migration involved in sprouting angiogenesis |
|  | xenobiotic detoxification | **miR-215** | interleukin-21 production |
| **miR-145** | epithelial to mesenchymal transition |  | gene silencing |
|  | cell migration | **miR-218** | cell population proliferation |
|  | cellular response to hypoxia |  | cell migration |
| **miR-148b** | gene silencing |  | collagen biosynthetic process |
| **miR-150** | gene silencing |  | gene silencing |
|  | endothelial cell differentiation |  | MAP kinase activity |
|  | cell migration involved in sprouting angiogenesis |  | vascular associated smooth muscle cell migration |
|  | mesodermal cell differentiation |  |  |
