# Supplementary material for: Changes in miRNA expression in the lungs of pigs supplemented with different levels and forms of vitamin D
Source: Mol Biol Rep. 2023 Dec 12;51(1):8. doi: 10.1007/s11033-023-08940-1 (PMC10716066; doi:10.1007/s11033-023-08940-1)
Supplement: Supplementary file 4 — Supplementary Material 4: Table S2. Results of qPCR validation of mRNA sequencing data of pigs supplemented with different doses and forms of vitamin D [file 11033_2023_8940_MOESM4_ESM.docx]

|  |  | **miR-381-3p** | | **miR-215** | | **miR-96-5p** | |
| --- | --- | --- | --- | --- | --- | --- | --- |
| **Group** | **Samples** | **counts** | **RQ^[[1]](#footnote-1)^** | **counts** | **RQ** | **counts** | **RQ** |
| **1** | a10 | 5070,844 | 1 | 165,070 | 1 | 261,261 | 1 |
|  | a17 | 3674,881 | 0,394 | 394,166 | 0,711 | 88,928 | 0,258 |
|  | a18 | 2995,600 | 0,788 | 122,764 | 0,224 | 64,413 | 0,22 |
|  | a34 | 2751,363 | 0,585 | 100,310 | 0,503 | 315,260 | 0,691 |
|  | a41 | 44509,601 | 3,137 | 255,549 | 0,498 | 220,301 | 2,057 |
|  | a42 | 4160,123 | 0,659 | 354,579 | 0,855 | 257,689 | 0,343 |
|  | a9 | 845,261 | 0,203 | 673,925 | 2,871 | 33,125 | 0,064 |
| **2** | b11 | 1796,707 | 0,261 | 1451,424 | 1,356 | 27,808 | 0,038 |
|  | b12 | 7638,665 | 1,206 | 118,217 | 0,488 | 184,904 | 0,425 |
|  | b19 | 1568,688 | 0,367 | 314,010 | 0,515 | 113,071 | 0,37 |
|  | b20 | 3368,166 | 0,412 | 283,148 | 1,856 | 304,430 | 0,636 |
|  | b27 | 4521,760 | 0,826 | 9974,655 | 3,202 | 69,748 | 0,362 |
|  | b36 | 2703,441 | 0,504 | 328,401 | 0,739 | 48,869 | 0,23 |
|  | b44 | 11225,251 | 0,328 | 807,183 | 0,89 | 112,958 | 0,314 |
| **3** | c14 | 837,220 | 0,59 | 527,015 | 1,265 | 105,625 | 0,453 |
|  | c21 | 1114,644 | 0,181 | 528,329 | 1,369 | 56,146 | 0,147 |
|  | c29 | 2059,046 | 0,301 | 413,175 | 3,014 | 121,790 | 0,252 |
|  | c30 | 1580,733 | 0,086 | 932,764 | 0,959 | 57,446 | 0,204 |
|  | c38 | 1260,505 | 0,204 | 457,779 | 0,332 | 90,266 | 0,397 |
|  | c53 | 586,854 | 0,244 | 99,209 | 0,336 | 81,362 | 0,488 |
| **4** | d16 | 1135,054 | 0,324 | 619,404 | 1,344 | 149,000 | 0,571 |
|  | d23 | 44558,023 | 0,397 | 24525,614 | 3,519 | 129,473 | 0,383 |
|  | d24 | 3048,071 | 0,948 | 1306,836 | 2,431 | 124,374 | 0,52 |
|  | d32 | 2593,865 | 0,16 | 479,359 | 0,812 | 153,395 | 0,386 |
|  | d56 | 4009,518 | 0,623 | 255,127 | 0,638 | 103,393 | 0,3 |
|  | d63 | 14555,201 | 2,242 | 372,353 | 0,653 | 225,560 | 1,535 |
|  | d7 | 3118,014 | 0,52 | 99,917 | 0,604 | 430,939 | 0,846 |
|  | **r2** | **0,604** | | **0,631** | | **0,581** | |

**Table S2.** Results of qPCR validation of mRNA sequencing data of pigs supplemented with different doses and forms of vitamin D.

1. Relative Quantification [↑](#footnote-ref-1)
